# A cluster-randomized trial of the effectiveness of a triple-faceted intervention promoting adherence to primary care physician visits by diabetes patients

**DOI:** 10.1038/s41598-020-59588-x

**Published:** 2020-02-18

**Authors:** Mitsuhiko Noda, Yasuaki Hayashino, Katsuya Yamazaki, Hikari Suzuki, Atsushi Goto, Masayuki Kato, Kazuo Izumi, Masashi Kobayashi

**Affiliations:** 1 Department of Diabetes, Metabolism and Endocrinology, Ichikawa Hospital, International University of Health and Welfare, 6-1-14 Kounodai, Ichikawa, Chiba Japan; 2 Diabetes Research Center, National Center for Global Health and Medicine, 1-21-1 Toyama, Shinjuku-ku, Tokyo Japan; 3 Department of Endocrinology, Tenri Hospital, 200 Mishimacho, Tenri, Nara Japan; 4 Department of Epidemiology and Healthcare Research, Kyoto University Graduate School of Medicine, Yoshida-konoe-cho, Sakyo-ku, Kyoto Japan; 5 First Department of Internal Medicine, Faculty of Medicine, Toyama University, 2630 Sugitani, Toyama, Toyama Japan; 6 Kawai Clinic, 715-1 Higashihiratsuka, Tsukuba, Ibaraki Japan; 7 Japan Community Health care Organization Takaoka Fushiki Hospital, 8-5 Fushiki Kofumotomachi, Takaoka, Toyama Japan; 8 Epidemiology and Prevention Group, Center for Public Health Sciences, National Cancer Center, 5-1-1 Tsukiji, Chuo-ku, Tokyo, Japan; 9 Toranomon Hospital Health Management Center and Diagnostic Imaging Center, 2-2-2 Toranomon, Minato-ku, Tokyo Japan; 10 Center for Clinical Sciences, National Center for Global Health and Medicine, 1-21-1 Toyama, Shinjuku-ku, Tokyo Japan

**Keywords:** Type 2 diabetes, Health care

## Abstract

We aimed to assess whether a triple-faceted intervention program administered in the primary care setting could decrease the risk of insufficient adherence to primary care physician (PCP) appointments among this patient population. We conducted a cluster-randomized controlled study to assess the effects of a 1-year intervention. The primary outcome was insufficient adherence to regular PCP attendance for diabetes treatment, defined as failure to visit a PCP within 2 months of an original appointment date. The intervention consisted of mailing patient reminders of their PCP appointments, providing patients with health education aimed at lifestyle modification and benchmarking PCP procedures. Eleven municipal level district medical associations employing 192 PCPs were divided into two subregions for assignment to intervention and control clusters, with 971 and 1,265 patients assigned to the intervention and control groups, respectively. Primary outcome data were available for 2,200 patients. The intervention reduced insufficient adherence to regular PCP appointments by 63% (hazard ratio, 0.37; 95% confidence interval [CI], 0.23–0.58). In conclusion, a triple-faceted intervention program consisting of health education, appointment reminders, and physician benchmarking may decrease the risk of incomplete adherence to regular PCP appointments by diabetes patients.

## Introduction

The incidence and prevalence of type 2 diabetes are rapidly increasing worldwide^1–4^, including in Japan, where a national survey estimated that the number of patients with diabetes had increased from 6.9 to 9.5 million between 1997 and 2012^5,6^. This rate of increase is a significant concern because epidemiologic studies have indicated that type 2 diabetes is an independent risk factor for cardiovascular diseases as well as microvascular complications. In support of this indication of the potential broad-reaching impact of diabetes, the incidence of cardiovascular disease is reported to be approximately twice as high in patients with diabetes as in healthy age-matched individuals^7^. In response to these findings, researchers and clinicians have underscored the need for intensive control of glycemic response and lipid and blood pressure levels to reduce risk of cardiovascular disease and microvascular complications in patients with diabetes^8^.

Although clinical guidelines recommend both nonpharmacological and pharmacological interventions to control diabetes-associated complications^9^, patients cannot benefit from these interventions if they are lost to follow-up. The current rate of adherence to diabetes treatment in Japan is approximately 80–90%^10,11^, which is similar to the estimates in the United States, Scotland, Canada^12^, and United Kingdam^13^. Patients who do not regularly visit their primary care physicians (PCPs) tend to have poorer glycemic and obesity control^14–16^, both of which may lead to poor health outcomes that ultimately result in the development of diabetic complications^17^. Furthermore, nonadherence to diabetes treatment and relapse after temporary improvement may explain a substantial amount of uncontrolled diabetes^18^. Strengthening the healthcare provider–patient relationship and performing certain administrative actions, such as calling or mailing reminders to patients prompting them to make or keep appointments, could increase clinic attendance and subsequently increase glycemic monitoring and disease control for these patients^8,14,15^. However, to our knowledge, there are no published reports on whether provision of a multifaceted intervention program in the primary care setting that includes these components reduces the treatment dropout rate of diabetes patients in a real-world clinical setting. To address this research gap, the Japan Diabetes Outcome Intervention Trial 2 (J-DOIT2) investigated whether provision of a triple-faceted intervention program in the primary care setting could decrease the risk of insufficient adherence to regular PCP appointments by patients that have been diagnosed with type 2 diabetes and tested the hypothesis that intervention efficacy will vary according to specific patient characteristics.

## Methods

### Ethics statement

This study was approved by Ethics Committee of the Office of Strategic Outcomes Research Program, Japan Foundation for the Promotion of International Medical Research Cooperation, in accordance with the Declaration of Helsinki and relevant ethical guidelines in Japan. All participants received a precise explanation of the study and provided their written informed consent.

### Study design

Specific details regarding the study participants and the methods used in this 1-year, prospective, cluster-randomized interventional study have been described elsewhere (Trial Registration: UMIN000002186, https://upload.umin.ac.jp/cgi-open-bin/ctr_e/ctr_view.cgi?recptno = R000002663; date of registration: July 13, 2009)^19^. In brief, PCPs within each district medical association (DMA) in Japan were divided into two groups according to the geographical location of the PCP’s clinic and the DMA branch to which the PCP belonged; neighboring clinics belonging to the same DMA branch were included in the same group. Therefore, clusters formed were composed of PCPs belonging to the same DMA branches and their patients with diabetes.

### DMAs and PCPs

To be eligible for the study, a DMA needed to have approximately 20 PCPs who were able to participate in the study. We estimated that the study required 125 patients with diabetes from each DMA. In addition, the DMA had to be capable of establishing a diabetes treatment network consisting of PCPs, physicians specializing in diabetes, physicians specializing in kidney disease, and ophthalmologists. The PCP eligibility criteria were as follows: a membership to a recruited DMA, being a nonspecialist in the treatment of diabetes, and a realistic capability of enrolling 10 or more consenting patients with diabetes to the study. PCPs who had participated in a study with similar interventions during the preceding 5 years were excluded. Fifteen municipal level DMAs in Japan were recruited through an announcement made by the Japan Medical Association, a nationwide professional organization representing physicians throughout Japan. From this pool, 192 PCPs at 11 DMAs consented to participate in the study.

### Patients

The patient inclusion criteria were as follows: a diagnosis of type 2 diabetes prior to registration and aged 40–64 years at enrollment. As we aimed to focus on generations who tend to actively work and have relatively high diabetes prevalence, the age groups of 40–64 years were selected. The exclusion criteria included undergoing hemodialysis, hospitalization, bedridden condition, nursing home residence, blindness, amputated lower limbs, diagnosis of a malignant tumor within the preceding 5 years, pregnancy or potential pregnancy, having two or more medical doctors in charge of diabetes care (except for the treatment of diabetes complications), and type 1 diabetes. There was no study restriction on plasma glucose level or glycated hemoglobin A1c (HbA1c) value.

### Randomization

After registration of the 11 DMAs, each DMA was divided into two subregions or clusters. The study statistician, blinded to the cluster of each DMA, used statistical software to randomly allocate a code of 0 (control) or 1 (intervention) to each of the subregions within each of the 11 DMAs. The other assignments were then made with stratification by DMA, such that each DMA was composed of one control cluster and the remaining as intervention clusters.

### Data collection

Clinical research coordinators (CRCs) obtained study data from each patient’s medical chart. Patients in both groups were asked to complete a self-administered questionnaire pertaining to their lifestyle and diabetes-related distress. Distress was measured using the Problem Areas in Diabetes (PAID) scale. CRCs visited each PCP clinic to collect follow-up data, including laboratory results, dates of completed patient appointments, and patients’ next appointment dates.

### Interventions

The interventions consisted of sending reminders regarding their regular PCP appointments and providing patients with health education aimed at lifestyle modification via a treatment support center that had been established for these purposes beforehand. Reminders for regular medical visits consisted of a letter sent 1 week before the established next visit day (NVD). If this appointment was missed, another letter was sent 2 weeks after the NVD. If required, a telephone call was made to the patient 4 weeks after the NVD. If the patient did not visit the PCP within 6 weeks of the NVD, the PCP or a clinic staff member contacted the patient by either letter or telephone.

Lifestyle modification intervention was provided to encourage progress toward behavioral changes in diet and exercise. Certified diabetes educators, registered dieticians, or public health nurses participating in the standardized program for behavioral theory on patient education provided counseling, which was tailored for each patient according to his or her PCP’s instructions with regard to the target body weight, recommended food intake, and exercise therapy. Patients received six sessions of lifestyle advice through telephone calls, each lasting between 15 and 30 min. Alternatively, some DMAs trained certified diabetes educators and provided them with a location for face-to-face counseling. With these DMAs, there were four face-to-face advice sessions, each lasting approximately 30 min. Furthermore, the PCPs in the intervention group received feedback letters regarding the indicators for themselves as well as the benchmarks for each indicator. All interventions continued for 1 year.

### Outcome measures

The primary study outcome was adherence to regular PCP visits for diabetes treatment. Non-adherence was defined as failure to attend follow-up appointments regularly, that is, failure to visit a PCP within 2 months of the original appointment. The time to the first non-adherence to attend the PCP was defined as the primary outcome measure. The study’s secondary outcome measures were patients’ HbA1c level, random blood glucose level, body mass index (BMI), and systolic and diastolic blood pressure. Other prespecified outcome measures include quality of diabetes treatment, patients’ behavioral stages of change, and patients’ clinical data such as lipid profile and body weight^19^. Among them, results of quality of diabetes treatment have been previously published^20^.

### Sample size estimation

Based on the main outcome parameter of insufficient adherence to regular PCP appointments, a sample size estimation was established via a power calculation performed according to the method reported by Hayes and Bennett^21^. As moderate variations in cluster size have little impact on the sample size estimation^21^, we further assumed that all clusters are of equal size. Based on the results of the previously completed J-DOIT2 pilot study, the effect size of the intervention was estimated to be a 45% reduction in insufficient adherence, an incidence of the primary outcome measure of 71.6 per 1,000 person-years, and a coefficient of variation of 0.43. Based on these assumptions, a two-tailed alpha of 5%, and a beta of 10%, it was estimated that if 125 patients were recruited per cluster, 15 clusters per arm would be needed, requiring a sample of at least 3,750 patients.

### Statistical analysis

The characteristics of patients in the intervention and control groups were calculated as proportions, means, and standard deviations. Because randomization was at the cluster level, patient-level characteristics were not likely to be as balanced as they would be if randomization had been performed at the patient level. We examined the balance of covariates using fixed-effects linear regression for continuous confounders (e.g., age) and fixed-effects binomial or multinomial logistic regression for categorical confounders (e.g., gender), with consideration of clustering within each DMA. Adherence to regular visits was analyzed as the time from entry into the study until the first event, that is, the first missed appointment. Patient data were censored at the end of the follow-up period for this trial (1 year). To test the study hypothesis, we used a Cox proportional hazard model that included a Huber–White sandwich estimator for clustered data. Primary data were analyzed using the intention-to-treat principle. Next, to test the hypothesis that the effectiveness of the intervention varied according to individual patient characteristics (age group [40–59 vs. 60–65 years], gender [male vs. female], HbA1c level [<8% vs. ≥8%], working status [working vs. nonworking], and PAID quartiles), we conducted a stratified analysis of these covariates. The HbA1c data were converted to equivalent values of the National Glycohemoglobin Standardization Program according to a statement made by the Japan Diabetes Society^22^.

We used linear regression, taking clustering into account, to estimate the association between the intervention and secondary outcomes (HbA1c level, random blood glucose level, systolic and diastolic blood pressure, and BMI). The measure of effect was the mean difference in outcomes derived by subtracting the baseline value from that of the last observation between the intervention and control groups. All statistical tests were two sided with an alpha level of 0.05. All analyses were performed using Stata/MP version 12.0 (Stata Corporation, College Station, TX, USA).

## Results

**Figure 1:**
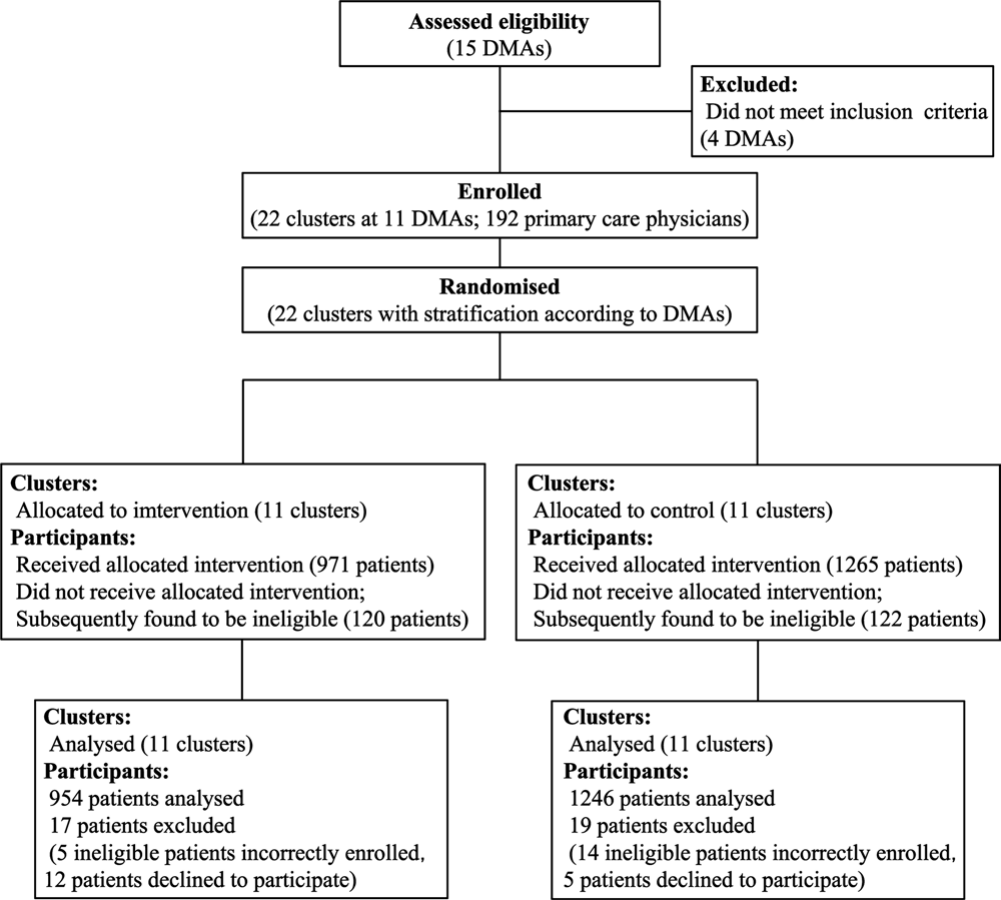
Flow chart of study cluster enrollment and patient selection procedures. DMAs, district medical associations.

Figure 1 summarizes this trial’s allocation of study clusters and eligible patients. Study participants were recruited between July 2009 and September 2009. After an intervention and a control group were established within each of the 11 eligible DMAs by random assignment, all 1,091 and 1,387 patients in the collective intervention and control groups, respectively, were assessed for study eligibility. From these groups, 971 and 1,265 patients who were determined to be eligible were assigned to the intervention and control groups, respectively. All 22 clusters were followed for 1 year (until October 2010).

Patient baseline characteristics are presented in Table 1. The mean patient age was 56.5 years; 37.5% were women, and their mean HbA1c level was 7.4%, with no significant differences between the intervention and control groups with one exception: patients in the intervention group were more likely to be administered diabetes medication of any kind (*P* = 0.049). Protocol compliance was 86.8% (5,097 of 5,872) for reminders sent 1 week before the scheduled NVD, 28.9% (486 of 1,680) for those sent 2 weeks after the NVD, 48.2% (143 of 331) for those sent 4 weeks after the NVD, and 76.7% (46 of 60) for those sent 6 weeks after the NVD. On an average, patients in the intervention group received 4.4 sessions on lifestyle modification.

**Table 1:** Baseline characteristics* of participants.

Characteristic	Total	Control	Intervention	*P* value
	*n* = 2,200	*n* = 1,246	*n* = 954	
Age, years	56.5 (5.9)	56.5 (5.9)	56.5 (5.9)	0.935
Female, %	37.5	36.3	39.1	0.108
BMI, kg/m^2^	26.0 (4.2)	26.0 (4.1)	25.9 (4.3)	0.533
HbA1c, %	7.4 (1.3)	7.4 (1.2)	7.4 (1.3)	0.334
Diabetes therapy, %				0.049
No medication	10.6	12.0	8.9	
Oral hypoglycemic agent only	81.2	80.1	82.6	
Insulin	8.2	8.0	8.5	
Work, %	77.0	77.3	76.7	0.742
PAID	36.0 (13.1)	36.5 (13.4)	35.2 (12.7)	0.128

*Results are presented as the mean (standard deviation) unless otherwise indicated.

BMI, body mass index; HbA1c, glycated hemoglobin A1c; PAID, problem area in diabetes scale.

**Figure 2:**
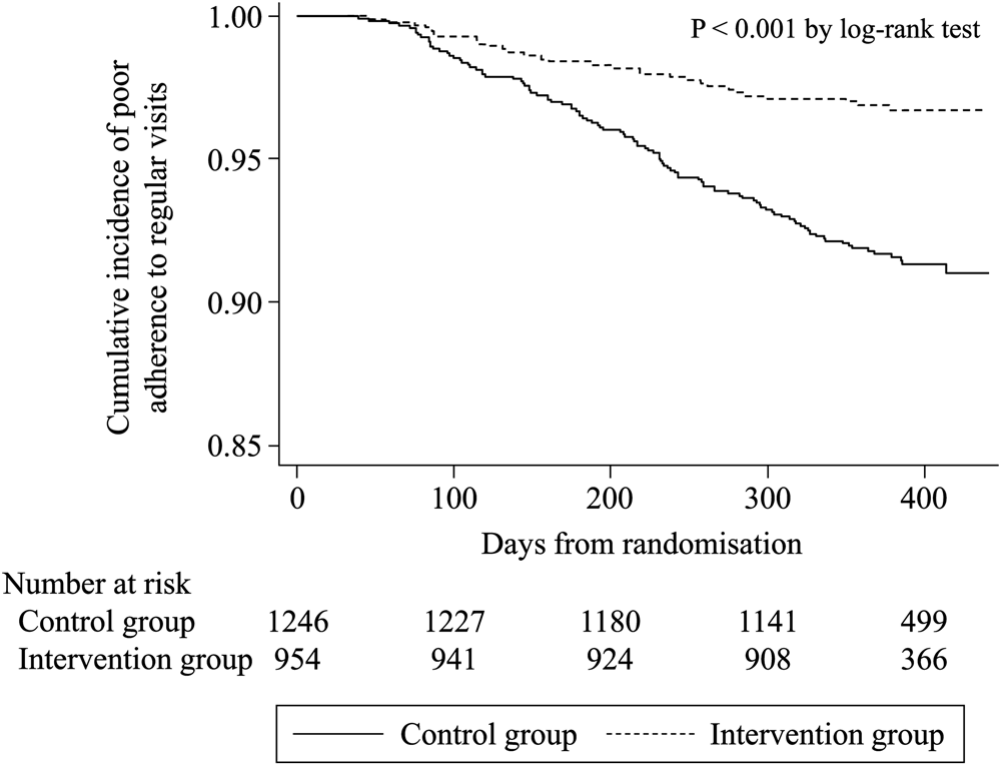
Kaplan–Meier estimates of insufficient adherence to primary care attendance by patients with diabetes. Solid line: control group; broken line: intervention group.

During a median follow-up period of 1.1 years, there were 135 patients with insufficient adherence to PCP visits. As illustrated by the Kaplan–Meier curves for the intervention and control groups (Fig. 2), the primary study outcome of a reduction in the incidence of insufficient adherence to regular attendance was significantly different between the intervention and control groups (*P* < 0.001; log-rank test). Specifically, the frequencies of insufficient adherence were 82.5 per 1,000 person-years and 30.4 per 1,000 person-years in the control and intervention groups, respectively (Table 2). The estimated hazard ratio, defined here as the risk of insufficient adherence by the intervention group vs. the control group, was 0.37 (95% confidence interval [CI], 0.23–0.58; *P* < 0.001). This significant effect of intervention persisted even after adjustment for the type of diabetes therapy.

**Table 2:** Effect of the intervention on primary and secondary outcomes.

	Control	Intervention	*P* value			
	(*n* = 1,246)	(*n* = 954)				
***Primary outcome***						
Person-years	1,272	987				
No. of events	105	30				
Incidence (per 1,000 person-years)	82.5	30.4	<0.001			
HR (95% CI)	0.37 (0.23–0.58)		<0.001			
Adjusted HR (95% CI)*	0.38 (0.24–0.59)		<0.001			
	**Control**		**Intervention**		**Difference in change from baseline to follow-up (95% CI)**	***P*** **value**
	**Baseline**	**Follow-up**	**Baseline**	**Follow-up**		
Secondary outcomes**						
HbA1c, %	6.9 (1.2)	6.8 (1.1)	7.0 (1.3)	6.7 (1.1)	−0.17 (−0.27 to −0.07)	0.004
Random blood glucose, mg/dl	150.1 (57.3)	154.2 (63.3)	151.2 (58.7)	146.9 (53.2)	−8.15 (−11.29 to −5.03)	<0.001
Systolic blood pressure, mmHg	132.0 (14.8)	132.5 (15.7)	130.5 (14.1)	130.0 (14.2)	−0.89 (−2.66 to 0.89)	0.292
Diastolic blood pressure, mmHg	78.2 (10.2)	77.8 (10.3)	76.6 (9.4)	76.1 (9.3)	0.14 (−0.90 to 1.19)	0.761
BMI, kg/m^2^	26.0 (4.2)	26.1 (4.3)	25.9 (4.3)	25.7 (4.3)	−0.21 (−0.33 to −0.10)	0.002

*Adjusted for diabetes medication use.

**Data are means (standard deviations) unless otherwise indicated.

BMI, body mass index; HbA1c, glycated hemoglobin A1c; HR, hazard ratio.

**Figure 3:**
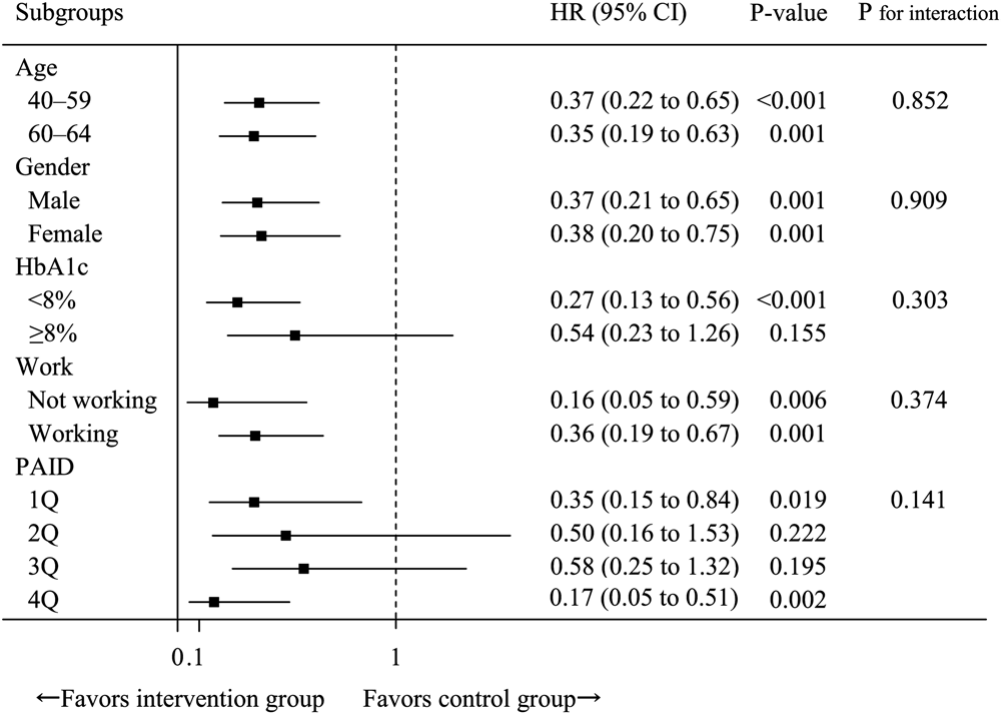
Effect of intervention on the primary outcome stratified according to the baseline characteristics. Black squares: point estimates of the hazard ratio; bars: confidence intervals. PAID, problem areas in diabetes scale.

Table 2 and Fig. 3 summarize the analysis results stratified according to subgroup. These results showed that the effect of intervention was not significantly modified by age, gender, extent of glycemic control, employment status, or diabetes distress as measured by the PAID scale. Patients in the intervention group experienced significant reductions in HbA1c level (−0.17%; 95% CI, −0.27 to −0.07; *P* = 0.004), random blood glucose level (−8.15 mg/dl; 95% CI, −11.29 to −5.03; *P* < 0.001), and BMI (−0.21; 95% CI, −0.33 to −0.10; *P* = 0.002), but not in systolic (*P* = 0.292) or diastolic (*P* = 0.761) blood pressure.

## Discussion

To date, very few data have been reported that support the strategy of providing interventions as an effective method for increasing patient adherence to a treatment regimen^23^. One example of a positive outcome was from a before-after study, which suggested a benefit from providing information on appointments, mainly by a telephone call^24^. To our knowledge, our study is the first cluster-randomized controlled trial to evaluate the effectiveness of a triple-faceted intervention for improving the adherence to regularly scheduled primary care visits by diabetes patients. Our results showed that such a coordinated intervention reduced the risk of insufficient adherence to regular primary care visits in patients with diabetes by 63%.

A plan for continuing care is an essential feature in the management of patients with diabetes. Clinical guidelines recommend regular visits be scheduled for better diabetes care^25^; however, controlled clinical trials addressing adherence to regular visits by patients with diabetes are rare. Prior to our study, only one reported patient-level randomized controlled trial had attempted to address this issue^26^. This previous study evaluated 854 noninstitutionalized patients with diabetes who were more than 15 years old and were treated with insulin or oral hypoglycemic agents. These patients had attended a hospital outpatient department in Indianapolis within the previous year and had another scheduled appointment on record. The intervention was similar to the one presented here: patients were provided reminders of appointments, and if a patient missed his or her appointment, he or she was contacted again by telephone or letter. However, the overall reduction in the number of missed appointments in that study was not statistically significant. In contrast, our study showed that, in our patient population, insufficient adherence to regular visits is an almost entirely correctable problem.

There are several possible explanations for the difference in outcomes between the two studies. First, as shown in previous research, the quality of the patient–provider relationship is associated with patient adherence to diabetes treatment^27^. In this regard, lifestyle modification intervention, one of the components of our study, was offered to subjects based on their specific stages according to the transtheoretical model of behavior change^28^. This approach may have helped establish good healthcare provider–patient relationships, which in turn may have improved patient adherence to their regular PCP appointments. Second, the previous study was a patient-level randomized controlled trial, whereas our study used a cluster-randomized controlled trial design, which reduced the contamination of the intervention effect among study participants. Third, the difference in the study settings may have also influenced the effectiveness of the intervention. Our study was conducted in the primary care setting rather than in the hospital, which may help explain the difference in efficacy, which might have facilitated the ability to improve the patient experience and their adherence to the intervention.

We found that the combination of patient education with appointment reminders and benchmarking of PCP procedures was effective in maintaining adherence to regular clinic visits, although this result needs to be interpreted cautiously. In a subgroup analysis, the effect of the intervention was not different between different age groups, but this result does not guarantee that the intervention will be effective in age groups other than those included in this trial. Furthermore, we did not include patients younger than 40 years, since the intervention was ineffective in this age group during an earlier phase of the trial. In fact, intervention tended to worsen the adherence in this generation, although the difference was not statistically significant^29^. Additional studies are required to investigate other methods of improving the adherence of younger patients to continuous diabetes care.

This trial also showed that education on lifestyle modification was effective in improving HbA1c level, random plasma glucose level, and BMI, but not for blood pressure control. Previous studies have produced contradictory results on the effectiveness of lifestyle modification intervention in improving blood pressure. The Turin study^30^, a 5-year randomized controlled trial that evaluated the effectiveness of structured group education programs in patients with diabetes, showed that such programs are effective in reducing BMI and HbA1c levels and improving patient quality of life but not for improving fasting plasma glucose levels. However, this study involved a relatively small number of patients (*n* = 120).

Insufficient adherence to regular PCP appointments might have a direct influence on clinical outcomes because it reduces the continuity of care, resulting in missed opportunities to check for comorbidities or titrate medications; delays appropriate interventions and referrals to specialists by PCPs; and hinders the development of a good healthcare provider–patient relationship^31^. Development of a sustained, reliable relationship may influence both patient and physician behaviors in a manner that would improve self-care and glucose control. From the patient perspective, a good relationship may improve their sense of trust in their PCP. As this relationship improves, patients may become more comfortable in divulging critical information pertaining to their social context that is relevant to their health and healthcare. This information may improve physician decision-making concerning patient’s disease management and could contribute to the effectiveness of the intervention on secondary outcomes.

This study has several strengths. First, it evaluated a large number of patients and included DMAs located throughout Japan. Second, the intervention was designed to be implemented in the primary care setting. Third, the trial had a robust cluster design that effectively reduced contamination between practices. There are several limitations that merit to discuss. First, the intervention and control groups were not well matched with respect to the type of diabetes therapy being administered. However, such an imbalance is not uncommon in cluster randomized controlled trials^32^. Moreover, the significant effect of the intervention persisted even after adjusting for this difference. Second, our sample size did not reach the goal based on the sample size calculation, possibly resulting in insufficient power and thus imprecise findings. However, we observed a statistically significant risk reduction probably because the effect size was much larger than we expected.

In conclusion, this study demonstrates that a triple-faceted intervention consisting of healthcare education aimed at modifying lifestyle and an appointment reminder system with benchmarking of PCP procedures can significantly decrease poor patient compliance and improve patient adherence to regularly scheduled PCP appointments. Because it is unclear if specific aspects of the intervention were responsible for this outcome, future research should attempt to identify the efficacy of the individual components of intervention. Widespread application of the intervention program evaluated in this study may result in a large decrease in the patient dropout rate from participation in regular diabetes treatment, a key to the prevention of long-term diabetic complications^33^, potentially leading to large reductions in medical costs.

## Data Availability

To comply with our privacy and data security policies, the data of current study are available only for researchers who meet our criteria for access to confidential data. For researchers who have an interest in using the data, please contact the corresponding author.
