# Author Correction: A cluster-randomized trial of the effectiveness of a triple-faceted intervention promoting adherence to primary care physician visits by diabetes patients: J-DOIT2 large-scale trial (J-DOIT2-LT)

**DOI:** 10.1038/s41598-020-70072-4

**Published:** 2020-10-19

**Authors:** Mitsuhiko Noda, Yasuaki Hayashino, Katsuya Yamazaki, Hikari Suzuki, Atsushi Goto, Masayuki Kato, Kazuo Izumi, Masashi Kobayashi

**Affiliations:** 1 Department of Diabetes, Metabolism and Endocrinology, Ichikawa Hospital, International University of Health and Welfare, 6-1-14 Kounodai, Ichikawa, Chiba Japan; 2 Diabetes Research Center, National Center for Global Health and Medicine, 1-21-1 Toyama, Shinjuku-ku, Tokyo, Japan; 3 Department of Endocrinology, Tenri Hospital, 200 Mishimacho, Tenri, Nara, Japan; 4 Department of Epidemiology and Healthcare Research, Kyoto University Graduate School of Medicine, Yoshida-konoe-cho, Sakyo-ku, Kyoto, Japan; 5 First Department of Internal Medicine, Faculty of Medicine, Toyama University, Sugitani, Toyama, Toyama 2630 Japan; 6 Kawai Clinic, 715-1 Higashihiratsuka, Tsukuba, Ibaraki Japan; 7 Japan Community Health Care Organization Takaoka Fushiki Hospital, 8-5 Fushiki Kofumotomachi, Takaoka, Toyama Japan; 8 Epidemiology and Prevention Group, Center for Public Health Sciences, National Cancer Center, 5-1-1 Tsukiji, Chuo-ku, Tokyo, Japan; 9 Toranomon Hospital Health Management Center and Diagnostic Imaging Center, 2-2-2 Toranomon, Minato-ku, Tokyo, Japan; 10 Center for Clinical Sciences, National Center for Global Health and Medicine, 1-21-1 Toyama, Shinjuku-ku, Tokyo, Japan

Correction to: *Scientific Reports* https://doi.org/10.1038/s41598-020-59588-x, published online 18 February 2020

The original version of this Article contained an error in the Title where “: J-DOIT2 large-scale trial (J-DOIT2-LT)” was omitted.

Additionally, the Acknowledgements section in the original version of the Article was incomplete.

“The authors thank Dr. Maki Goto for her helpful support in preparing the manuscript; Dr. Ryotaro Bouchi for his helpful advice in preparing the revised manuscript. This work was supported by grants from the Japan Agency for Medical Research and Development (Practical Research Project for Life-Style related Diseases including CVD and Diabetes); the Ministry of Health, Labour and Welfare of Japan (Strategic Outcomes Research Program for Research on Diabetes; Comprehensive Research on Life-Style Related Diseases including CVD and Diabetes H25-016); and the Japan Society for the Promotion of Science KAKENHI (Grant‐in‐Aid for Scientific Research) grant number 15H04779. The funding source had no role in study design, data collection, data analysis, data interpretation, the writing of the report, or the decision to submit the article for publication.”

now reads:

“The authors thank Dr. Maki Goto for her helpful support in preparing the manuscript; Dr. Ryotaro Bouchi for his helpful advice in preparing the revised manuscript. We also appreciate the support of the J-DOIT2 study group including the members of the district medical associations of Shimotsuga County and City (Tochigi Prefecture), Chiba City (Chiba Prefecture), Itabashi Ward (Tokyo Metropolis), Iida (Nagano Prefecture), Takaoka City (Toyama Prefecture), Motosu (Gifu Prefecture), Omihachiman City and Gamo County (Shiga Prefecture), Yodogawa Ward of Osaka City (Osaka Prefecture), Tokushima City (Tokushima Prefecture), Kokura of Kitakyushu City (Fukuoka Prefecture), and Naha City (Okinawa Prefecture). This work was supported by grants from the Japan Agency for Medical Research and Development (Practical Research Project for Life-Style related Diseases including CVD and Diabetes); the Ministry of Health, Labour and Welfare of Japan (Strategic Outcomes Research Program for Research on Diabetes; Comprehensive Research on Life-Style Related Diseases including CVD and Diabetes H25-016); and the Japan Society for the Promotion of Science KAKENHI (Grant‐in‐Aid for Scientific Research) grant number 15H04779. The funding source had no role in study design, data collection, data analysis, data interpretation, the writing of the report, or the decision to submit the article for publication.”

These errors have now been corrected in the PDF and HTML versions of the Article.

